# How Semantic Radicals in Chinese characters Facilitate Hierarchical Category-Based Induction

**DOI:** 10.1038/s41598-018-23281-x

**Published:** 2018-04-03

**Authors:** Xiaoxi Wang, Xie Ma, Yun Tao, Yachen Tao, Hong Li

**Affiliations:** 1 School of Preschool Education and Special Education, Kunming University, Kunming, 650214 China; 2 Key Laboratory of Educational Informatization for Nationalities, Yunnan Normal University, Ministry of Education, Kunming, 650500 China; 3 School of Educational Science and Management, Yunnan Normal University, Yunnan Sheng, China; 4 Martin de Tours School of Management and Economics, Assumption University of Thailand, Bangkok, Thailand; 5 Institute for Advanced Study, ChengDu University, Chengdu, 610106 China

## Abstract

Prior studies indicate that the semantic radical in Chinese characters contains category information that can support the independent retrieval of category information through the lexical network to the conceptual network. Inductive reasoning relies on category information; thus, semantic radicals may influence inductive reasoning. As most natural concepts are hierarchically structured in the human brain, this study examined how semantic radicals impact inductive reasoning for hierarchical concepts. The study used animal and plant nouns, organized in basic, superordinate, and subordinate levels; half had a semantic radical and half did not. Eighteen participants completed an inductive reasoning task. Behavioural and event-related potential (ERP) data were collected. The behavioural results showed that participants reacted faster and more accurately in the with-semantic-radical condition than in the without-semantic-radical condition. For the ERPs, differences between the conditions were found, and these differences lasted from the very early cognitive processing stage (i.e., the N1 time window) to the relatively late processing stages (i.e., the N400 and LPC time windows). Semantic radicals can help to distinguish the hierarchies earlier (in the N400 period) than characters without a semantic radical (in the LPC period). These results provide electrophysiological evidence that semantic radicals may improve sensitivity to distinguish between hierarchical concepts.

## Introduction

A multitude of studies examining the relationship between language and thought (see^1^ for more summative details) has concluded that language can affect thought, as advocated by the weak version of the Sapir-Whorf hypothesis, from several perspectives, especially in terms of the temporal world^2,3^ spatial world^4,5^, colour terms^6,7^, and even the category labels of a given language^8^. In natural categories, items form a hierarchical structure whereby each level has a different degree of abstraction, with greater abstraction at higher levels^9^. Usually, humans organize natural categories into subordinate (e.g., apple), basic (e.g., fruit), and superordinate (e.g., plant) levels. An important function of categories is to support inferences^10^. Inductive inferences go beyond the available data in a fundamental way and arrive at conclusions that are likely, but not certain, given the available evidence^11,12^. Inductive inference is closely related to linguistic information: research has revealed that linguistic information facilitates the acquisition of superordinate-level but not basic-level categories^13^. Other research has found that nouns facilitate superordinate classification, but render subordinate classification more difficult^14^. Studies of children and adults alike have found that a superordinate level label (e.g., animal) leads to improved short- and longer-term memory recall^15^.

Research into natural category recognition has found that language affects classification processing. For example, both Mandarin Chinese and English can provide explicit labels that denote category membership. In studies of English-speaking children, an explicit cue like *oak tree* can facilitate children’s learning of nouns and their categories better than cues without explicit category information, such as *oak*^16^. Words containing such labels are relatively rare in English, but they are highly prevalent in Mandarin Chinese^17,18^. Questions such as “Is plutonium/zinc a kind of metal?” might not be answerable by British and American English speakers without a science background; however, this is not necessarily the case with Chinese speakers. Chinese characters for metal objects contain a semantic radical (“”) denoting the metal category *per se*. Nearly 80% of commonly used Chinese characters have a pictophonetic structure (i.e., they contain semantic and phonetic radicals), 71% of which have pictophonetic characters that conform to the rule that the semantic radical provides category information, and the phonetic radical provides pronunciation information (these data were based on a study of 7,000 commonly used Chinese characters^19^). Regarding the aforementioned question, plutonium (“”) and zinc (“”) are both pictophonetic characters. They contain the same semantic radical (i.e., “”), and can be easily judged as metal objects even if an individual does not understand the exact meaning of the character. In Mandarin Chinese, the semantic radical serves as a category label for all category levels. For instance, the semantic radical “” labels metal objects. This semantic radical applies to category members at both the subordinate and the basic levels, which clearly indexes category membership. Even as young as 4-years-old, Chinese children can understand the category membership indexed by a semantic radical^20^.

Studies have shown that a semantic radical can facilitate categorization for single-^21^ and two-character words^22^. Words with semantic radicals garner shorter reaction times than those without semantic radicals. In addition, the above study also found that semantic radicals facilitated the reorganization of atypical items, whereby faster responses were obtained to atypical items with semantic radicals than to typical items without semantic radicals. Electrophysiological evidence from a semantic-classification task, wherein participants judged the membership of atypical (e.g., train) versus typical (e.g., car) pictorial exemplars of a category (e.g., vehicle), showed that English speakers exhibited larger N400 event-related potential (ERPs) component differences between typical and atypical members, whereas Mandarin speakers showed no such differences for morphologically transparent nouns (e.g., the morpheme “vehicle” *che1* in the noun “train” *huo3che1* ). However, orthographically transparent items elicited moderate N300 and N400 effects (e.g., the radical “bug” *chong*2 in the character for the noun “butterfly” *hu*2*die2* ). Thus, the semantic radical did not engender facilitation in this ERP study, which contradicts the behavioural study of Zhang and Peng^21^. However, in the behavioural study, the variable was the semantic radical; the researchers compared Chinese words with and without semantic radicals. In contrast, in the ERP study, the variables were the language and type of transparency. That is, English and Chinese were compared as morphologically transparent and orthographically transparent. Thus, whether the semantic radical can facilitate category processing as indicated by ERPs remains unclear.

The retrieval of category information from semantic radicals is closely related to the processing of Chinese characters. Chinese characters are first represented at the feature level, passed to the radical level where the radicals are combined into complex characters, and finally the intended concept can be accessed. Additionally, the semantic radical can allow retrieval of the concept information independently^23^. Therefore, the representation of a radical at the radical level, and the representation of that radical at the concept level are reciprocally linked. A semantic radical can be represented not only at the lexical level, but it can also allow independent access to the conceptual level^24^. Generally, a skilled reader develops word representation that includes strong interconnections among the three lexical constituents; that is, orthography, phonology, and semantics^25,26^. When a character contains a semantic radical, it generates a processing bias: semantic radicals correctly pertaining to a character’s meaning facilitate reaction times in a semantic categorization task, and the semantic path appears to be the default means of character recognition^27^. ERP evidence for semantic radical processing has revealed that, compared to a condition without a semantic radical, a larger N400 is found in a “with-semantic-radical” condition than in a “without-semantic-radical” condition. The greater N400 elicited by a Chinese character with a high-frequency radical can be interpreted in terms of impeded processing due to inhibition from a greater radical-neighbourhood^28^. Another study has also regarded the N400 as an index for impeded character processing^29^. The N400 component was first found in a sentence comprehension study, which indicated that its magnitude is modulated by semantic relationships, with larger N400 magnitudes occurring for semantic anomalies^30^. A larger N400 is found when processing pictophonetic characters, suggesting that more cognitive resources are needed when processing pictophonetic characters than when processing single characters.

Previous studies have revealed that ERP components, such as the N1, P3, N400, and LPC are sensitive to inductive reasoning, and the N1 is sensitive to selective attention, which influences the further processing of perceptual features^31^. Recent studies about reasoning have indicated that P3 amplitudes reflect the satisfaction of expectations. That is, the P3 amplitude was relatively large when tasks were undemanding, whereas more difficult demands usually reduced the P3 amplitude^32^. Late ERP components, such as the N400 and LPC, were also found for the processing of categorization tasks. Studies have shown that the N400 can also index category membership^33,34^, whereby typical members usually elicit a more moderate negative wave than atypical members. This suggests that the clearer the relationship, the smaller the N400 magnitude. During reasoning, the LPC component is modulated by the strength of a premise and conclusion; the LPC component is larger in amplitude in a mismatching-premise condition than in a matching-premise condition^35^.

Semantic radicals can allow access to concept-level information independent of category information retrieval, and there is evidence that characters with semantic radicals facilitate categorization compared to those without semantic radicals. Accordingly, we hypothesized that characters with semantic radicals (i.e., pictophonetic characters) would facilitate inductive reasoning regarding hierarchical concepts, and the facilitation would be closely related to the processing of pictophonetic characters. However, when considering the processing of pictophonetic characters, confusion has emerged. On the one hand, existing evidence (both behavioural and ERP data) has shown that pictophonetic characters facilitate categorization and reasoning compared to single characters^21,22^; on the other hand, there is evidence that more effort is required to process pictophonetic characters than to process single characters^28,29^. Combining these two sides together, it is difficult to discern how pictophonetic characters facilitate inductive reasoning in the former studies. Moreover, linguistic information is more closely related to superordinate-level concept processing in studies of syllabic languages. Whether the same is true of logographic languages, such as Chinese, remains unclear.

Therefore, the main purpose of this study was to clarify how semantic radicals facilitate category-based induction. We hypothesized that semantic radicals would facilitate hierarchical category-based inductive reasoning. In detail, a with-semantic-radical condition was expected to elicit faster reaction times and a higher accuracy rate. As semantic radicals provide physical similarity, a larger N1 amplitude may be elicited. Based on the aforementioned evidence that more difficult demands usually reduce P3 amplitudes, a lower P3 was expected to be elicited in the with-semantic-radical condition. As there is evidence that semantic radicals can facilitate the categorization, a lower LPC amplitude was expected in the with-semantic-radical condition. However, as there is conflicting evidence regarding the N400 component, it is difficult to infer the situation. As characters were used as the material in this study, the induction was based on linguistic processing: thus, in accordance with the results of linguistic studies, a larger N400 amplitude was expected in the with-semantic-radical condition.

To test this hypothesis, an inductive reasoning task was employed in which a subordinate-level concept was used as a premise, and the result could be the subordinate-, basic-, or superordinate-level concept. ERPs were recorded to investigate the electrophysiological underpinnings because this would also help confirm the detailed processing time periods.

## Results

### Behavioural responses

#### Reaction times

Main effects of the levels *F*(2, 34) = 7.441, *p* = 0.003, η_*p*_^2^ = 0.304, and semantic radicals *F*(1, 17) = 6.480, *p* = 0.021, η_*p*_^2^ = 0.271 were found, with participants reacting faster in the with-semantic-radical condition than in the without-semantic-radical condition. A post-hoc test (Bonferroni) of the levels showed that subordinate-subordinate reasoning elicited slower reactions than the subordinate-basic (*p* = 0.024) and subordinate-superordinate (*p* = 0.002) reasoning. There was no interaction between the levels and semantic radicals *F*(2, 34) = 0.735, *p* = 0.461, *η*_*p*_^2^ = 0.041.

#### Accuracy

**Figure 1:**
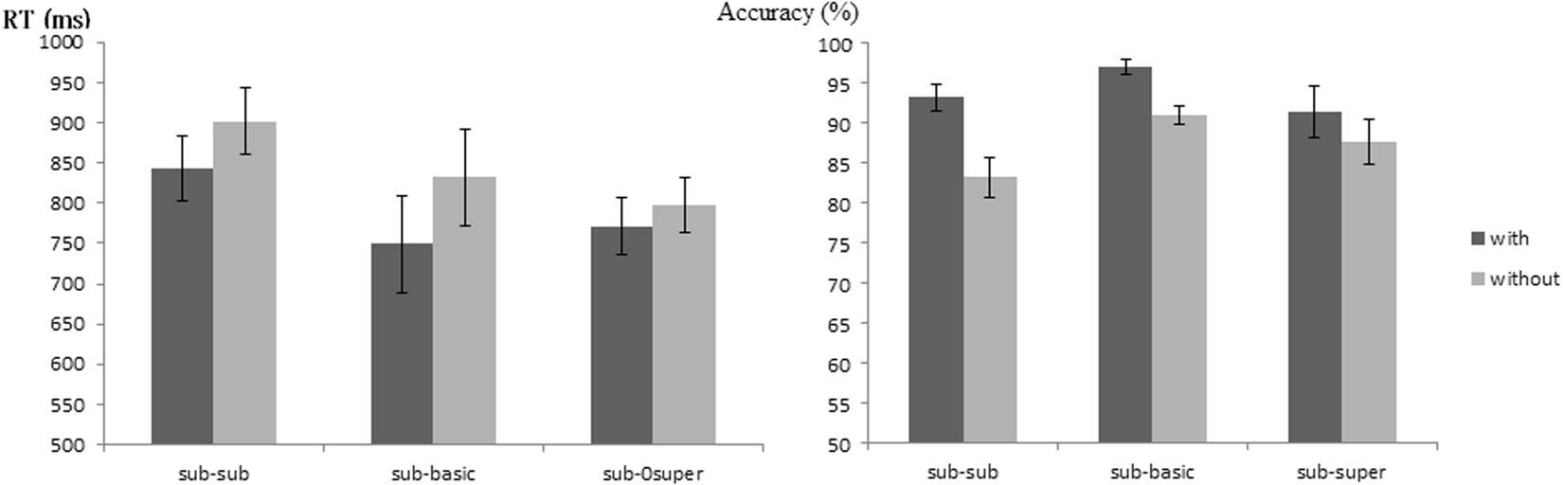
The mean reaction time and accuracy.

A main effect of the levels *F*(2, 34) = 2.841, *p* = 0.095, η_*p*_^2^ = 0.143 was not found, but a main effect of the semantic radicals *F*(1, 17) = 56.738, *p* < 0.001, η_*p*_^2^ = 0.769, was found; participants were more accurate in the with-semantic-radical condition than in the without-semantic-radical condition. An interaction between the levels and semantic radicals was found *F*(2, 34) = 5.574, *p* = 0.016, η_*p*_^2^ = 0.247. A simple effects analysis showed that the main effect of semantic radicals was found in the subordinate-subordinate *F*(1, 17) = 42.492, *p* < 0.001, *η*_*p*_^2^ = 0.714, subordinate-basic *F*(1, 17) = 21.156, *p* < 0.001, η_*p*_^2^ = 0.554, and subordinate-superordinate reasoning *F*(1, 17) = 8.132, *p* = 0.011, η_*p*_^2^ = 0.324. Another simple effects analysis showed that the main effect of the levels emerged in the without-semantic-radical condition *F*(2, 16) = 11.225, *p* < 0.01, η_*p*_^2^ = 0.584, and a post-hoc test indicated that subordinate-basic induction was more accurate than the subordinate-subordinate induction (*p* < 0.001). No other significant results were found (see Fig. 1).

### ERP responses

#### N1 (90–150 ms)

**Figure 2:**
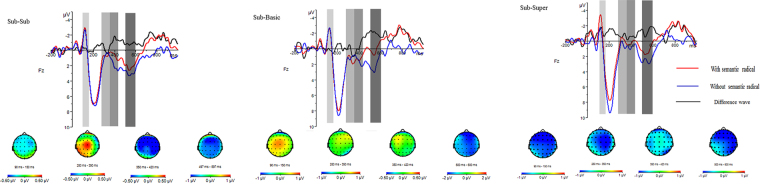
Waveforms and topographies elicited in sub-sub, sub-basic and sub-super reasoning.

**Figure 3:**
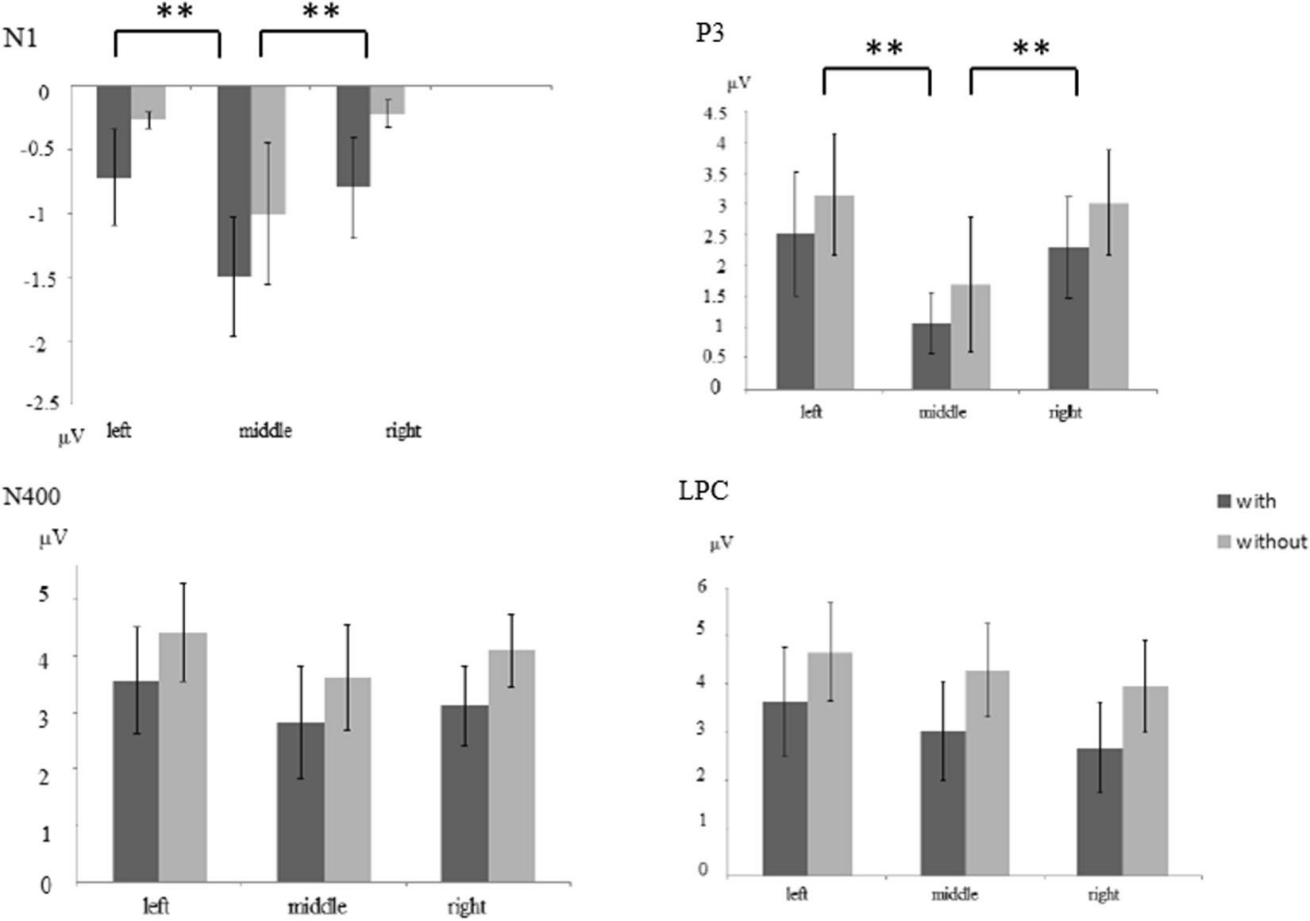
Bar figure of waveforms.

A main effect of the concept levels *F*(2, 34) = 2.599, *p* = 0.091, *η*_*p*_^2^ = 0.133 was not found, but a main effect was found for the semantic radicals *F*(1, 17) = 6.235, *p* = 0.023, η_*p*_^2^ = 0.268; a larger N1 amplitude was found in the with-semantic-radical condition. Main effects of frontality *F*(4, 68) = 4.475, *p* = 0.045, η_*p*_^2^ = 0.208 and laterality *F*(2, 34) = 11.145, *p* = 0.001, η_*p*_^2^ = 0.396 were found. A post-hoc test (Bonferroni) of frontality indicated that the frontal central (*p* = 0.023) and central sites (*p* = 0.009) elicited a larger negative amplitude than the parietal site. A post-hoc test (Bonferroni) of laterality also found that the middle line showed a larger negative amplitude than the left (*p* = 0.002) and right (*p* < 0.001) sides (see Figs 2 and 3).

#### P3 (280–350 ms)

A main effect of the concept levels *F*(2, 34) = 3.142, *p* = 0.059, *η*_*p*_^2^ = 0.156 was not found, but a main effect was found for the semantic radicals *F*(1, 17) = 4.770, *p* = 0.043, *η*_*p*_^2^ = 0.219, with a larger P3 amplitude found in the without-semantic-radical condition. Main effects of frontality *F*(4, 68) = 33.203, *p* < 0.001, η_*p*_^2^ = 0.661, and laterality *F*(2, 34) = 7.712, *p* = 0.003, η_*p*_^2^ = 0.312, were also found. A post-hoc test (Bonferroni) of frontality indicated that the frontal site showed a larger positive amplitude than the central site (*p* = 0.002), central parietal site (*p* < 0.001), and parietal site (*p* < 0.001). The central frontal site showed a larger positive amplitude than the central site (*p* < 0.001), central parietal site (*p* < 0.001), and parietal site (*p* < 0.001). The central site showed a larger positive amplitude than the central parietal (*p* < 0.001) and parietal sit (*p* < 0.001) site; and the central parietal site showed a larger positive amplitude than the parietal site (*p* = 0.019). A post-hoc test (Bonferroni) of laterality found that the middle line showed a lower positive amplitude than the left (*p* = 0.003) and right (*p* = 0.009) sides.

#### N400 (350–420 ms)

A main effect of the concept levels *F*(2, 34) = 1.911, *p* = 0.173, η_*p*_^2^ = 0.101 was not found, but a main effect was found for the semantic radicals *F*(1, 17) = 8.952, *p* = 0.008, η_*p*_^2^ = 0.249, with a larger N400 being found in the with-semantic-radical condition. An interaction between the concept levels and semantic radicals was found *F*(2, 34) = 12.766, *p* < 0.001, η_*p*_^2^ = 0.429, and a simple effects analysis showed that the main effect of the concept levels was found in the with-semantic-radical condition *F*(2, 16) = 4.431, *p* = 0.029, η_*p*_^2^ = 0.249, but not in the without-semantic-radical condition *F*(2, 16) = 0.507, *p* = 0.612, η_*p*_^2^ = 0.060. The post-hoc test (Bonferroni) of the with-semantic-radical condition indicated that subordinate-subordinate reasoning elicited a larger negative amplitude than the subordinate-basic (*p* = 0.011) and subordinate-superordinate (*p* = 0.016) reasoning. Another way of simple effects analysis found a difference of semantic radicals in the subordinate-subordinate (*p* < 0.001) and subordinate-superordinate reasoning (*p* < 0.034), larger N400 wave were found in the with-semantic-radical condition.

A main effect of frontality *F*(4, 68) = 114.828, *p* < 0.001, η_*p*_^*2*^ = 0.871 was found, but a main effect of laterality was not found *F*(2, 34) = 2.105, *p* = 0.143, η_*p*_^*2*^ = 0.110. A post-hoc test (Bonferroni) of frontality showed that the frontal site had a larger negative amplitude than the central frontal site (*p* = 0.001), central site (*p* < 0.001), central parietal site (*p* < 0.001), and parietal site (*p* < 0.001); the frontal central site showed a larger negative amplitude than the central site (*p* < 0.001), central parietal site (*p* < 0.001), and parietal site (*p* < 0.001); the central parietal site showed a larger negative amplitude than the parietal site (*p* < 0.001) and parietal site (*p* < 0.001); and the central parietal site showed a larger negative amplitude than the parietal site (*p* = 0.015).

#### LPC (500–600 ms)

Main effects of the concept levels *F*(2, 34) = 4.00, *p* = 0.034, η_*p*_^2^ = 0.190, and semantic radicals *F*(1, 17) = 9.626, *p* = 0.006, η_*p*_^2^ = 0.362 were found, with a larger positive amplitude in the without-semantic-radical condition. A post-hoc test (Bonferroni) of the concept levels found that the subordinate-subordinate reasoning elicited a larger positive amplitude than the subordinate-basic reasoning (*p* = 0.052).

A main effect of frontality *F*(4, 68) = 30.657, *p* < 0.001, η_*p*_^2^ = 0.643 was found, but this was not found for laterality *F*(2, 34) = 2.964, *p* = 0.091, η_*p*_^2^ = 0.148. A post-hoc test (Bonferroni) of frontality indicated that the frontal site showed a larger positive amplitude than the central site (*p* = 0.006), central parietal site (*p* < 0.001), and parietal site (*p* < 0.001); the frontal central site showed a larger negative amplitude than the central site (*p* < 0.001), central parietal site (*p* < 0.001), and parietal site (*p* < 0.001); and the central parietal site showed a larger negative amplitude than the parietal site (*p* < 0.001) and parietal site (*p* = 0.045). (Detailed see Table 1).

**Table 1:** Four-way repeated-measures ANOVA of mean amplitudes to assess the effects of semantic radicals at three concept levels. *Indicates significant at the 0.05 level. **Indicates significant at the 0.01 level.

	N1			F	P3	*η* ^2^	N400			LPC		
	F	*p*	*η* ^2^		*p*		F	*p*	*η* ^2^	F	*p*	*η* ^2^
Frontality	4.475	**0.045**	0.208	33.203	**0.000**	0.661	114.83	**0.000**	0.871	30.66	**0.000**	0.643
Laterality	11.164	**0.001**	0.396	7.712	**0.003**	0.312	2.105	0.143	0.11	2.964	0.091	0.148
Levels	2.599	0.091	0.133	3.142	0.059	0.156	1.911	0.173	0.101	4.000	**0.034**	0.19
Semantic radical	6.235	**0.023**	0.268	4.77	**0.043**	0.219	8.952	**0.008**	0.345	9.626	**0.006**	0.362
Frontality* Laterality	9.133	**0.000**	0.349	3.222	**0.028**	0.159	2.131	0.099	0.114	8.444	**0.001**	0.332
Frontality* Levels	0.413	0.683	0.024	7.91	**0.001**	0.318	10.854	**0.000**	0.39	1.527	0.229	0.082
Laterality* Levels	1.574	0.217	0.085	3.362	**0.038**	0.165	0.981	0.398	0.055	1.306	0.282	0.071
Frontality* Laterality* levels	1.549	0.185	0.084	1.614	0.166	0.087	1.761	0.133	0.094	1.617	0.178	0.087
Frontality* semantic radical	4.466	0.033	0.208	1.246	0.298	0.068	2.815	0.093	0.142	0.942	0.383	0.052
Laterality* semantic radical	0.447	0.575	0.026	0.303	0.639	0.018	1.467	0.246	0.079	2.323	0.141	0.12
Frontality* Laterality * Semantic radical	1.151	0.34	0.063	1.146	0.341	0.063	0.368	0.817	0.021	0.842	0.501	0.047
Levels* semantic radical	2.401	0.107	0.124	2.247	0.139	0.117	12.766	**0.000**	0.429	1.632	0.218	0.088
Frontality* Levels* semantic radical	0.765	0.458	0.043	2.246	0.090	0.117	4.282	**0.014**	0.201	0.749	0.518	0.042
Laterality*Level* semantic radical	0.449	0.645	0.026	1.817	0.167	0.097	1.096	0.352	0.061	0.490	0.663	0.028
Frontality*Laterality*Levels*semantic radical	1.037	0.405	0.057	0.912	0.484	0.051	1.546	0.187	0.083	0.923	0.479	0.052

### Timing of the concept level effect in the with- and without-semantic-radical conditions

The above results imply that the concept level effect occurred earlier in the with-semantic-radical condition (e.g., N400, 350–420 ms) than in the without-semantic-radical condition (e.g., LPC, 500–600 ms). In order to test the reliability of this time effect, we conducted a separate analysis of variance (ANOVA) of the timing (N400 and LPC), concept level effect, and semantic radical effect. As the main purpose of the test was to examine the timing effect, the electrode position effect was not taken into consideration. A three-way interaction between timing, concept level, and semantic radical was found *F*(2, 68) = 3.221, *p* = 0.046, η_*p*_^2^ = 0.087. A simple effects analysis found that for the with-semantic-radical condition, the concept level effect occurred in the N400 time period *F*(2, 34) = 6.111, *p* = 0.009, η_*p*_^2^ = 0.264, the subordinate-subordinate inference elicited a larger N400 amplitude than the subordinate-basic (*p* = 0.011) and subordinate-superordinate (*p* = 0.016) inferences, and there was no concept level effect in the LPC period *F*(2, 34) = 2.120, *p* = 0.145, η_*p*_^2^ = 0.110. For the without-semantic-radical condition, the concept level effect was not found in the N400 time period *F*(2, 34) = 0.877, *p* = 0.396, η_*p*_^2^ = 0.049, but it did occur in the LPC time period *F*(2, 34) = 5.833, *p* = 0.0126, η_*p*_^2^ = 0.255.

## Discussion

In the present study, a main effect of concept level was found for the reaction times but not for accuracy. Post-hoc tests indicated that subordinate-subordinate inferences required the longest reaction time. This result may be due to the similarity of the subordinate concepts leading to a longer time requirement to discriminate between them.

More importantly, the main effect of a semantic radical was significant for both reaction time and accuracy. Characters with a semantic radical engendered faster reaction times and greater accuracy, which indicates that semantic radicals facilitated inductive inferences in terms of behavioural performance. Furthermore, an interaction was present between the concept level and semantic radical for accuracy. In the without-semantic-radical condition, a main effect of levels was found. The subordinate-basic level showed the highest accuracy, while no main effect of level emerged in the with-semantic-radical condition, which suggests that semantic radicals reduced the difference between the three levels. For the behavioural data, it can be concluded that semantic radicals facilitated the category-based induction in all three hierarchies.

Regarding the ERPs, for the N1, P3, N400, and LPC, a main effect of semantic radicals was found in all time periods. The N1 component indicates spatially selective attention, which is modulated by covert orienting such that a greater amplitude is observed for attended areas than for unattended areas^36^. During the N1 time window, the characters were processed at the feature level, and a larger N1 was found in the with-semantic-radical condition, indicating that greater attentional resources were captured by the semantic radicals. In this study, characters with semantic radicals have more strokes than those without, which may indicate that the more complex a character is, the more attention it attracts. The N1 wave in our study showed a frontal-central distribution, which could originate from the motor cortex, supplementary motor area, and/or cingulate gyrus^37^.

A previous study found that more difficult demands usually reduce the P3 amplitude^32^, and the lower P3 amplitude elicited in the with-semantic-radical condition may indicate that more effort was needed when recognizing pictophonetic characters than recognizing those with a semantic radical. This may be related to the processing of pictophonetic characters; unlike the phonetic-semantic method of syllabic languages, logograph characters, like those used in Chinese, employ a special picture-semantic method. Compared to characters without a semantic radical, pictophonetic characters involve a more complex processing period in which the semantic radical and phonetic radical are first separated at the radical level to retrieve its information. They are then combined at the character level to access the actual meaning, while characters without a semantic radical are processed as a feature and then transferred to the character level. In addition, fMRI studies of Chinese character processing have found that intrinsic orthographical information may also require additional orthographic-to-semantic mapping to be accessed. As such, more need more semantic processing of characters containing semantic radicals is required compared with those without semantic radicals. Thus, P3 can serve as an index of selective attention^38^. The parietal distribution of the P3 component in our study should be regarded as a task-relevant P3b component^39^. The P3b originates when temporal-parietal mechanisms process stimulus information for memory storage^40^, and the amplitude of the P3 elicited by a stimulus is predictive of later memory for that stimulus^41^. Thus, the smaller P3b for the characters with a semantic radical reflects the increased attention consumption for information retrieval from memory for the category.

The N400 component was first found in a sentence comprehension study, which indicated that its magnitude is modulated by semantic relationships, with larger N400 magnitudes occurring for semantic anomalies^42^. Moreover, other studies showed that the N400 could also index category membership^33,34^. These results suggest that the clearer the relationship is, the smaller the N400 magnitude should be. In the present study, an interaction between the semantic radicals and concept levels was found during the N400 time period, indicating that the semantic radicals and concept levels impact inductive reasoning in a combined fashion. The simple effects analysis showed that a larger N400 amplitude of the with-semantic-radical characters was found for subordinate-subordinate and subordinate-superordinate reasoning, while no effect was shown in the subordinate-basic reasoning. This may be because the basic-level concept has processing priority^9,13^. The larger N400 observed in the with-semantic-radical condition may be due to the processing of pictophonetic characters. Wu *et al*.^28^ found that the greater N400 elicited by Chinese characters with a high-frequency radical (all radicals in the present study are high-frequency radicals) can be interpreted in terms of impeded processing due to inhibition from a greater radical-neighbourhood. Another study also regarded the N400 as an index for impeded character processing^27^. Thus, the larger N400 found in the with-semantic-radical condition may relate to Chinese character processing; because characters with semantic radicals are more complex than those without, more cognitive resources may be required during semantic information retrieval.

The LPC component is modulated by the strength of a premise and conclusion; the LPC component is larger in amplitude in a mismatching-premise condition than in a matching-premise condition^35^. In the present study, a larger LPC was found in the without-semantic-radical condition, which means that participants may have lower confidence for a without-semantic-radical conclusion. The LPC component showed a posterior distribution. A previous study has revealed that a more posterior distribution of the LPC/P600/SPS indicates a parsing failure and/or an attempt to revise the syntactic structure^43^.

The timing analysis clearly shows that, in the with-semantic-radical condition, the concept level effect was observed during the N400 time window, while it emerged during the P600 time window in the without-semantic-radical condition. This finding means that a semantic radical can help to distinguish the hierarchical category concepts earlier. As the behavioural data showed that characters with semantic radicals enjoyed faster reaction times and a higher accuracy rate, this indicates that the semantic radical actually facilitated inductive reasoning. As the inductive reasoning task in our study was based on hierarchical category concepts, the mechanism underlying facilitation may be that the earlier the hierarchy can be distinguished, the easier/faster induction can be.

The facilitation of inferences by semantic radicals is closely related to the activation of semantic information processing, as shown in the radical representation model^23^. A semantic radical is first processed at the feature level, wherein its physical features are recognized. This is the perceptual processing stage, which is indicated by the N1 component, wherein characters with semantic radicals capture more attentional resources and thus elicit a larger N1.

The second stage is processing at the radical level. During this stage, semantic information spreads from the lexical network to the conceptual network. As the ERP evidence showed, during the P3 and N400 time periods, more effort was required for characters with a semantic radical, as there are more radicals in this condition than in the without-semantic-radical condition. As the N400 component relates to semantic relationships, the P3 time window may be an index of the information spread from the lexical network to the conceptual network, because increased information content in a pictophonetic character results in more difficult processing.

The third stage is the character level, in which not only semantic information but also category information is accessed. As the hierarchical conceptual levels are more abstract than the actual meaning of each word, and the recognition of the hierarchical levels means that the character has reached the conceptual level, not only can the actual meaning be accessed, but also the abstract level can be fully recognized. The recognition occurred earlier when a character had a semantic radical (N400 period) than when it did not (LPC period).

In summary, facilitation of inductive inferences by semantic radicals is closely related to the semantic radical information retrieval processes. Activation starts from the physical features, passes to the lexical network, and then spreads to the conceptual network, with different mechanisms at each stage. The semantic radical main effect lasted from the N1 period to the LPC period, while the category hierarchy effect only emerged from the N400 period. This may indicate that semantic radical information retrieval was earlier than category information, that category hierarchy processing is based on semantic information retrieval, and that the semantic radical can help to distinguish between the hierarchies earlier. Moreover, the combined behavioural and ERP results together demonstrate that a semantic radical can have a great impact on category-based induction in all three levels. The differences revealed by the characters with and without a semantic radical reflects how language shapes our thoughts; the facilitation caused by the semantic radical is not innate, but is gradually formed during the use of pictophonetic characters.

In conclusion, the physical and/or semantic features of semantic radicals facilitate the inductive inference of hierarchical concepts. Such facilitation is closely related to semantic radical processing as lexical information was accessed earlier than conceptual information. Semantic radicals facilitate category-based induction in all three hierarchies. Semantic radicals can help to distinguish between the hierarchies earlier than characters without a semantic radical.

## Methods

### Participants

Eighteen healthy undergraduate students (7 male, 11 female) participated in the study. All participants were right-handed with normal or corrected-to-normal vision, and they had no history of brain injury or mental illness. They had not taken part in similar experiments before. All received reimbursement for their participation after the experiment. The experiment was conducted in accordance with the ethical principles of the 1964 Declaration of Helsinki (World Medical Organization, 1996). All experimental protocols were approved by the University’s ethics committee (the Ethics Committee of Kunming University), and the methods were carried out in accordance with the relevant guidelines and regulations. All participants were fully informed of the experimental procedure and other requirements, and they signed an informed consent form before experiments commenced.

### Materials

In the experiment, two superordinate concepts (*animal* and *plant*) and six basic concepts were chosen (*bird*, *insect*, *mammal*, *vegetable*, *fruit*, and *flower*). The basic concepts were easily classified due to their high frequency of use. Participants within the same participant pool who did not take part in the experiment were asked to write down as many members as possible of each basic concept category. Then, another group of 40 participants rated the typicality and familiarity of the members generated using a 7-point scale, where 7 indicated “typical,” 4 indicated “uncertain,” and 1 indicated “atypical.” For each category, the top 10 words with and without a semantic radical were selected for the main study. The semantic radical for the bird category is “”, for the insect category is “”, for the flower category is “”, for the mammal category is “”, for the vegetable category is “”, and for the fruit category is “” and “”. Because there were few members of the insect and bird categories without a semantic radical, only eight members from the insect and bird categories each without a semantic radical were chosen, so that 60 members of categories with semantic radicals and 56 members of categories without semantic radicals were selected for the six basic categories. The words with a semantic radical differed significantly from those without a semantic radical (*t*(55) = 8.73, *p* < 0.01) in terms of their typicality rating, which could be due to material attributes. Semantic radicals relevant to the category were used for typical members, which were expected to facilitate the classification processes. The typicality of the two groups of materials was matched by deleting the 10 words with the highest score for semantic radicals and the six lowest words for those without semantic radicals. Thus, the words with and without semantic radicals were equal in number (i.e., 50, for a total of 100 words). A paired *t*-test indicated that the typicality did not differ between groups (*t*(49) = 1.692, *p* = 0.097). Furthermore, when preparing materials, we not only asked students to rate the typicality, but also the familiarity, using a 7-point scale, where 7 indicated very familiar while 1 meant not familiar at all. A simple t-test for familiarity showed that there was no difference between with-semantic and without semantic radical conditions (*t*(1,49) = 1.660, *p* = 0.103). A significant difference was found in stroke numbers (*t*(1,49) = 3.340, p = 0.002); there are more strokes in characters that have semantic radicals.

Before the experiment, participants were familiarized with the 100 categorized words in a learning phase (i.e., deciding whether a word belonged to one of the six categories), and they then participated in the classification test. In the classification test, the name of a subordinate level concept (e.g., apple) was shown, and then a basic level or a superordinate level name followed. Participants were asked if the former name was a member of the latter category. The learning phase lasted approximately 10–30 minutes. Only when the accuracy reached 90% did the participant proceed to the next stage. Participants repeated the learning phase until 90% accuracy was reached.

### Design and procedure

There were two independent variables: namely, hierarchy (subordinate-superordinate, subordinate-basic, and subordinate-subordinate) and semantic radical (with-semantic-radical and without-semantic-radical). The experiment used a 3 × 2 within-subjects design, with 720 trials in total. For each level of hierarchy, there were 240 trials, which consisted of 120 “yes” responses and 120 “no” responses. After each set of 80 trials, participants took a 5 minute break. The participants sat 60 cm from a screen (17” monitor, 85 Hz refresh rate) and were asked to fixate on its centre. The E-Prime 2.0 software and a hardware response box (SR-box; Psychology Software Tools Inc., Pittsburgh, USA) were used to present the stimuli.

At the beginning of each trial, a fixation cross was presented in the centre of a grey screen for 500 ms. Subsequently, a randomly chosen premise (a Chinese word) appeared in the centre of the screen for 650 ms, followed by a blank screen for 100 ms. Then, a capital letter, for example “X,” was presented in the centre of the screen for 100 ms, followed by a blank screen. The capital letter denoted that the premise had the characteristics of X (a capital on behalf of a character). Because six basic level categories were employed in the study, six letters were chosen (bird, insect, mammal, vegetable, fruit, and flower). The main purpose of the task was to perform inductive reasoning regarding whether a specific feature was shown (for example: “Does an apple contain vitamin C?” Then the participant was asked whether a pearl also contains vitamin C). Therefore, as participants could solve the inductive reasoning based on their knowledge rather than induction, abstract letters were chosen to represent a feature of each category.

**Figure 4:**
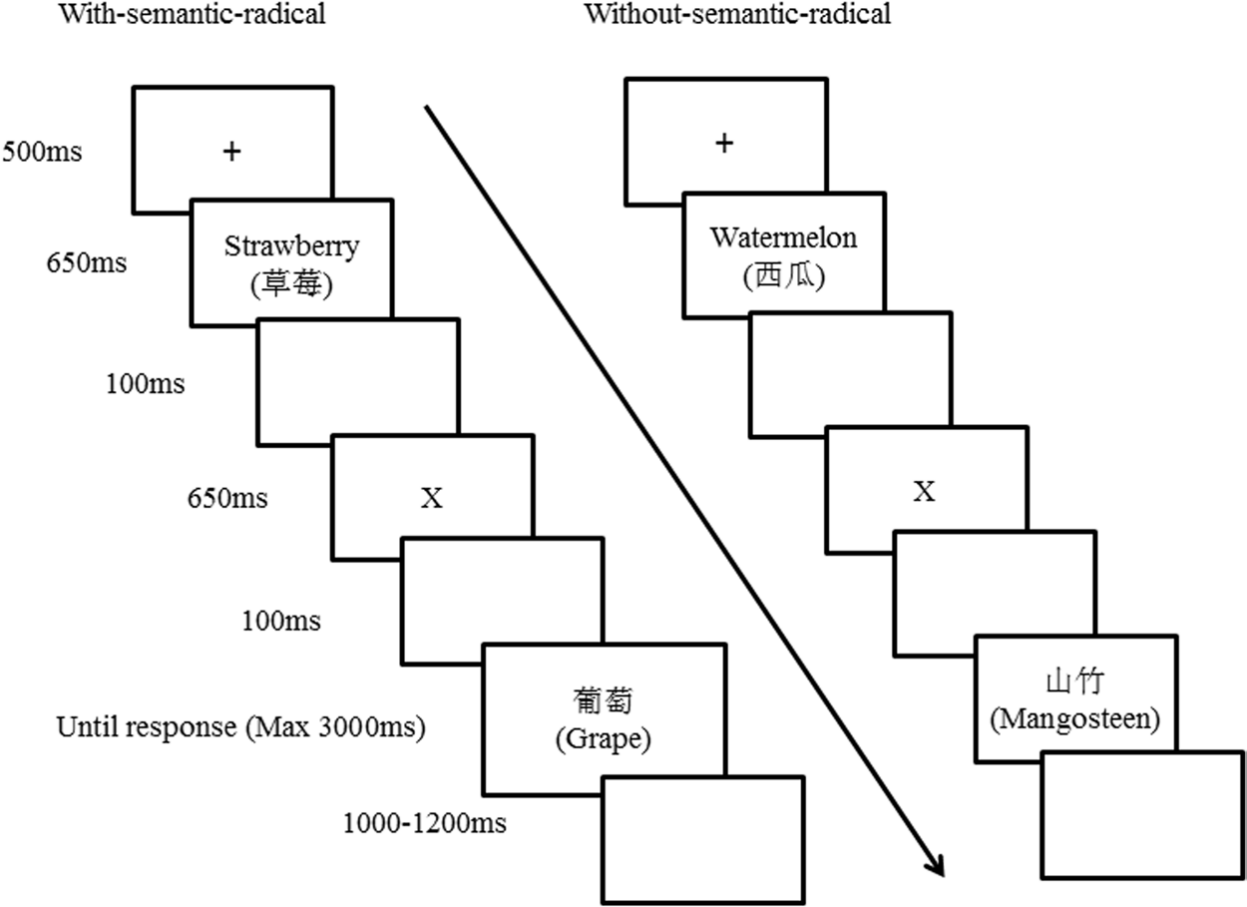
The design and procedure of the experiment.

Subsequently, the result was shown, and participants judged whether the results had the same character X as the premise. If no response was obtained after 3,000 ms, the experiment continued with the next trial. A response or no response was followed by a blank screen of 1,000–1,200 ms duration (see Fig. 4).

The instructions for the experiment were as follows: you will see a fixation cross at the centre of the screen. Subsequently, you will see the name of an object and then a capital letter, for example “X,” will be presented at the centre of the screen. The capital letter means that the premise object has the characteristics of X, and the X is standing for the character. After the capital letter, you will see another object’s name. Your task is to judge whether the latter object possesses the feature “X.” If you consider that the latter object has the feature “X,” press the “1” key. Otherwise, press the “5” key. Before the formal experiment, there is a practice session. After the practice, if you still have questions about the experimental requirements, you can seek help from the experimenter and repeat the practice. Please react as quickly and as accurately as possible.

During the practice session, when the participants performed the correct reasoning, they saw the mark “√” after the judgment (if the former word and the latter word were in the same category, it was judged as correct). There were different capital letters assigned to each category, “A” for bird, “B” for insect, “C” for mammal, “W” for vegetable, “X” for fruit, and “Y” for flower.

### ERP recordings and data analysis

Brain electrophysiological activity was recorded from 64 Ag/AgCl electrodes mounted on an elastic cap (Brain Products, Munich, Germany), with a ground electrode on the medial frontal line, using the FCz online reference and offline algebraic re-reference analysis to the left and right mastoids^39^. The impedance of all electrodes was maintained below 5 kΩ. The horizontal electrooculograms (EOGs) were recorded from the orbital rims of both eyes. The vertical EOG was recorded from the left eye both supraorbitally and infraorbitally. The signals were band-pass filtered (0.05–100 Hz) and continuously sampled at the 500 Hz/channel for offline analysis. Offline computerized artifact rejection software was used to eliminate trials with EOG artifacts (mean EOG voltage exceeding ± 80 μV). There were over 40 trials for each of the three levels of the hierarchy (subordinate-superordinate, subordinate-basic, and subordinate-subordinate), which meets the criteria for ERP analysis^44^. Repeated-measures ANOVAs were performed on the mean amplitudes within the time windows, with the factors of hierarchy (three levels: subordinate-superordinate, subordinate-basic, subordinate-subordinate), semantic radical (two levels: with-semantic-radical and without-semantic-radical), laterality (three levels: left, middle, and right sites), and frontality (five levels: frontal [left-F3, middle-Fz, right-F4]; frontal central [left-FC3, middle-FCz, right-FC4]; central [left-C3, middle-Cz, right-C4]; central parietal [left-CP3, middle-CPz, right-CP4]; and parietal [left-P3, middle-Pz, right-P4]). For all analyses, the degrees of freedom of the *F* ratio were corrected for violations of the sphericity assumption based on the Greenhouse-Geisser correction, and Bonferroni corrections were used for each comparison.

## References

[1] Pavlenko, A. The Sapir-Whorf Hypothesis and the bilingual turn in the study of language and cognition. *The bilingual mind: And what it tells us about language and thought*. 1–40 (Cambridge University Press, 2014).

[2] Friedman WJ (2004). Time in autobiographical memory. Social Cognition.

[3] Hannah, R. Time in Antiquity:an introduction. *Time in Antiquit*y. 1–5 (Routledge: London. 2009).

[4] Li P, Abarbanell L, Gleitman L, Papafragou A (2011). Spatial reasoning in Tenejapan Mayans. Cognition.

[5] Li P, Gleitman L (2002). Turning the tables: Language and spatial reasoning. Cognition.

[6] MacLaury, R. E. Color and Categorization. *Color and cognition in Mesoamerica: Constructing categories as vantage 379–392*. (University of Texas Press, 1997).

[7] Paramei, G. V. Russian ‘blues’. *Anthropology of color: Interdisciplinary multilevel modeling*, 75 (2007).

[8] Hupka RB, Lenton AP, Hutchison KA (1999). Universal development of emotion categories in natural language. Journal of personality and social psychology.

[9] Rosch E, Mervis CB, Gray WD, Johnson DM, Boyes-Braem P (1976). Basic objects in natural categories. Cognitive Psychol.

[10] Murphy GL (2003). The downside of categories. Trends Cogn Sci.

[11] Anderson, J. R. & Anderson, J. R. The adaptive character of thought: Erlbaum. *Automatica* (1990).

[12] Charles K, Joshua B (2009). T. Structured statistical models of inductive reasoning. Psychol Rev.

[13] Horton Marjorie S, Markman Ellen M (1980). Developmental Differences in the Acquisition of Basic and Superordinate Categories. Child Development.

[14] Waxman SR (1990). Linguistic biases and the establishment of conceptual hierarchies: Evidence from preschool children ☆. Cognitive Development.

[15] Gülgöz S, Gelman SA (2015). Children’s recall of generic and specific labels regarding animals and people. Cognitive Development.

[16] Gelman SA, Wilcox SA (1989). Conceptual and lexical hierarchies in young children. Cognitive Development.

[17] Zhou X, Marslen-Wilson W, Taft M, Shu H (1999). Morphology, Orthography, and Phonology in Reading Chinese Compound Words. Language & Cognitive Processes.

[18] Liu C (2010). What’s in a name? Brain activity reveals categorization processes differ across languages. Hum Brain Mapp.

[19] Li, Y., Kang, J., Wei, L. & Zhang, S. Research Of Moddern Chinese Pictophonetic Chracters. *Applied Linguistics* (1992).

[20] Dan, L. C. Q. L. Relationship between Categorization and Category-based Induction in Early Age. *Psychological Development & Education* (2008).

[21] Zhang, J. & Zhang, H. The recovery of meaning of chinese characters in the classifying process. *Acta Psychologica Sinica* (1990).

[22] Zhang J (1993). Experimental study on the retrieval of feature meaning of chinese words. Acta Psychologica Sinica.

[23] Ding G, Peng D, Taft M (2004). The nature of the mental representation of radicals in Chinese: a priming study. Journal of Experimental Psychology Learning Memory & Cognition.

[24] Chen XK, Zhang JJ (2012). Role of Familiarity of Semantic Radicals in the Recognition of Lowly Familiar Chinese Characters. Acta Psychologica Sinica.

[25] Perfetti C, Liu Y, Tan L (2005). The Lexical Constituency Model: Some Implications of Research on Chinese for General Theories of Reading. Psychol Rev.

[26] Tao, Y. & Liu, Y. study of word learning in minority nationality children. *A study on the Chinese Learning of the Children of Minority Nationalities*. 100–116, (China Sciences Publising & Media. Ltd, 2016).

[27] Williams C, Bever T (2010). Chinese Character Decoding: A Semantic Bias?. Reading & Writing An Interdisciplinary Journal.

[28] Wu Y, Mo D, Tsang YK, Chen HC (2012). ERPs reveal sub-lexical processing in Chinese character recognition. Neurosci Lett.

[29] Wang Q, Dong Y (2013). The N2- and N400-like effects of radicals on complex Chinese characters. Neurosci Lett.

[30] Kutas M, Hillyard SA (1980). Reading senseless sentences: Brain potentials reflect semantic incongruity. Science.

[31] Luck SJ, Woodman GF, Vogel EK (2000). Event-related potential studies of attention. Trends Cogn Sci.

[32] Polich J (2007). Updating P300: an integrative theory of P3a and P3b. Clin Neurophysiol.

[33] Fujihara N, Nageishi Y, Koyama S, Nakajima Y (1998). Electrophysiological evidence for the typicality effect of human cognitive categorization. Int J Psychophysiol.

[34] Nunez-Pena MI, Honrubia-Serrano ML (2005). N400 and category exemplar associative strength. Int J Psychophysiol.

[35] Bonnefond M, Castelain T, Cheylus A, Henst JBVD (2014). Reasoning from transitive premises: An EEG study. Brain & Cognition.

[36] Eimer M (1998). Mechanisms of Visuospatial Attention: Evidence from Event-related Brain Potentials. Visual Cognition.

[37] Giard MH (1994). Dissociation of temporal and frontal components in the human auditory N1 wave: a scalp current density and dipole model analysis. Electroencephalography and Clinical Neurophysiology/Evoked Potentials Section.

[38] Rushby JA, Barry RJ, Doherty RJ (2005). Separation of the components of the late positive complex in an ERP dishabituation paradigm. Clinical Neurophysiology Official Journal of the International Federation of Clinical Neurophysiology.

[39] Luck, S. J. A closer look at ERPs and ERP Components. *An introduction to the event-related potential technique* 49–98, (MIT press, 2014).

[40] Polich J, Criado JR (2006). Neuropsychology and neuropharmacology of P3a and P3b. Int J Psychophysiol.

[41] Wilding EL, Ranganath C (2011). Electrophysiological correlates of episodic memory processes. The Oxford handbook of ERP components.

[42] Kutas M, Hillyard SA (1984). Brain potentials during reading reflect word expectancy and semantic association. Nature.

[43] Kaan E, Swaab T (2003). Repair, revision, and complexity in syntactic analysis: An electrophysiological differentiation. Cognitive Neuroscience, Journal of.

[44] VanPatten, B. Input processing by novices–issues in the nature of processing and in research methods. *First Exposure to a Second Language: Learners’ Initial Input Processing*, 193 (Cambridge University Press 2014).

